# Measuring outcomes of the first trial of odour-baited mosquito traps for malaria control using a state of the art health and demographic surveillance system

**DOI:** 10.1186/1475-2875-13-S1-P97

**Published:** 2014-09-22

**Authors:** Tobias Homan, Alexandra Hiscox, Aurelio di Pasquale, Ibrahim Kiche, Collins Mweresa, Wolfgang Mukabana, Thomas Smith, Willem Takken, Nicolas Maire

**Affiliations:** 1 Laboratory of Entomology, Wageningen University, Wageningen, The Netherlands; 2 Department of Epidemiology and Public Health, Swiss Tropical and Public Health Institute, Basel, Switzerland; 3 International Centre of Insect Physiology and Ecology, Mbita, Kenya

## Background

The SolarMal project aims to eliminate malaria from Rusinga Island, western Kenya, by using the nationwide adopted strategy of malaria prevention (insecticide-treated bed nets and case management) augmented with mass trapping of mosquitoes. Mosquito traps, that emit human odour mimics to lure and trap malaria mosquitoes, are provided at the household level, with the intervention rolled out gradually to achieve mass coverage by mid-2015. Real time health and demographic data of the study population of 24,000 individuals are constantly being accumulated, and are instrumental in informing the study design and project logistics of SolarMal.

## Materials and methods

Our HDSS (Health and Demographic Surveillance System) is used to monitor population dynamics and malaria outcomes before and during the trial. We use an advanced and fully digitalized surveillance system and data management platform. OpenHDS (Open Health and Demographics System) is the first open source data management system to be designed for more efficient and cost effective data capture, management, and communication. Two cross-sectional malaria prevalence surveys of a representative sample of the population were conducted in 2012 and 2013. Malaria infections were diagnosed in the field using Rapid Diagnostic Test kits (RDTs). Baseline health and demographic data from 2012-2013 were analyzed to provide information about population demographics, malaria risk and spatial distribution of cases during this period.

## Results

Computer tablets with data collection software linked to the OpenHDS platform are shown to provide a comprehensive way to manage an HDSS and with this method yielding high quality data. The baseline distribution of malaria on Rusinga Island, during baseline, shows a malaria prevalence of 20.8% during the dry season, increasing to 26.9% in the wet season (N=2021 and N=1808). Individual, household, meteorological and geographical variables were found to be associated with malaria risk and possible spatial clustering of malaria was detected.

## Conclusions

Our HDSS systematically addresses the data collection and management challenges in HDSSs. It exploits modern client-server architectures and a mobile client for the Android platform which allows for centralized data management and point-of-capture digitization of data. Data gathered using this system are immediately available for analysis of population demographics and malaria risk.

